# Óxido Nítrico Sérico, Endotelina-1 Correlaciona Eventos Cardiovasculares Adversos Importantes Pós-Procedimento entre Pacientes com IAMCSST Agudo

**DOI:** 10.36660/abc.20240248

**Published:** 2024-12-16

**Authors:** Huihui Guo, Qi Qu, Jiechao Lv

**Affiliations:** 1 Department of Cardiovascular Medicine Shengzhou Hospital Shaoxing University China Department of Cardiovascular Medicine – Shengzhou People’s Hospital (Shengzhou Branch of the First Affiliated Hospital of Zhejiang University School of Medicine, the Shengzhou Hospital of Shaoxing University), Zhejiang – China

**Keywords:** Infarto do Miocárdio com Supradesnível do Segmento ST, Infarto do Miocárdio, Prognóstico

## Abstract

**Fundamento:**

O infarto do miocárdio com elevação do segmento ST (IAMCSST) é uma forma comum e grave de infarto agudo do miocárdio (IAM).

**Objetivos:**

O estudo teve como objetivo investigar a relação entre os níveis séricos de óxido nítrico (NO) e endotelina-1 (ET-1) com a gravidade do IAMCSST e seu valor preditivo para eventos cardiovasculares adversos maiores (MACE) dentro de um ano após intervenção coronária percutânea (ICP) em pacientes com IAMCSST.

**Métodos:**

O estudo retrospectivo foi conduzido em 269 pacientes com IAMCSST submetidos a ICP. Os pacientes foram categorizados em dois grupos: aqueles que desenvolveram MACE (112 casos) e aqueles que não desenvolveram (157 casos) em um ano. Os níveis de NO e ET-1 foram medidos no soro coletado usando ensaio imunoenzimático. A curva ROC ( *Receive-Operating Characteristics* ) foi usada para analisar o potencial prognóstico de NO e ET-1 individualmente e em combinação, p<0,05 foi considerado estatisticamente significativo.

**Resultados:**

Foram observadas diferenças significativas entre os dois grupos em relação à idade, classificação de Killip, fração de ejeção do ventrículo esquerdo, troponina I cardíaca (cTnI), creatina quinase-MB (CK-MB), bem como níveis séricos de NO e ET-1. O estudo observou que pacientes que desenvolveram MACE tinham níveis séricos mais baixos de NO e níveis mais altos de ET-1 na admissão. Análises posteriores revelaram uma relação inversa significativa entre os níveis séricos de NO e ET-1 e a gravidade do infarto do miocárdio. Um modelo de detecção combinado, -0,082 * NO + 0,059 * ET-1, demonstrou valor prognóstico promissor para a ocorrência de MACE dentro de um ano após a ICP.

**Conclusões:**

Os níveis séricos de NO e ET-1 servem como marcadores prognósticos valiosos para MACE em pacientes com STEMI submetidos a ICP, exibindo uma forte correlação com a gravidade do IAM.

## Graphical abstract

**Figura Central:**
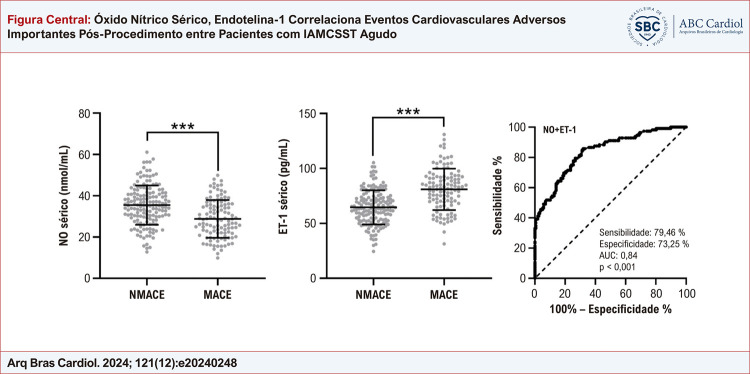
: Óxido Nítrico Sérico, Endotelina-1 Correlaciona Eventos Cardiovasculares Adversos Importantes Pós-Procedimento entre Pacientes com IAMCSST Agudo

## Introdução

O infarto agudo do miocárdio (IAM) é uma doença cardíaca comum em cardiologia, caracterizada pela redução ou interrupção repentina do fluxo sanguíneo devido à obstrução da artéria coronária,^1^ levando à hipóxia miocárdica, isquemia, necrose e complicações subsequentes, como dor no peito e arritmias.^2 , 3^ O início da doença é agudo, com progressão rápida, e pode representar um risco grave para a vida do paciente se não for prontamente intervindo. O IAM é categorizado em tipos como infarto do miocárdio com supradesnivelamento do segmento ST (IAMCSST), onda Q e alterações dinâmicas do segmento ST-T, sendo o IAMCST o mais prevalente.^4^ A ampla aplicação da intervenção coronária percutânea (ICP) de emergência reduziu significativamente a taxa de mortalidade de pacientes com IAMCSST.^5 , 6^ No entanto, isquemia prolongada e lesão de isquemia-reperfusão podem causar morte celular miocárdica, levando a eventos adversos cardiovasculares maiores (MACE) em curto prazo após o infarto do miocárdio, afetando gravemente o prognóstico do paciente. Portanto, marcadores de prognóstico de IAMCSST são críticos na redução da área de infarto do miocárdio, prevenindo a remodelação ventricular esquerda, reduzindo a ocorrência de MACE e, finalmente, melhorando o prognóstico de pacientes com IAMCSST.

As estratégias atuais de estratificação de risco para MACE abrangem sistemas de escores clínicos (por exemplo, escores GRACE, TIMI, PURSUIT),^7^ modalidades de imagem (ecocardiografia, angiografia coronária, ressonância magnética cardíaca (RM), angiotomografia computadorizada [TC]),^8 , 9^ e abordagens baseadas em biomarcadores.^10^ Enquanto biomarcadores cardíacos tradicionais, como troponinas cardíacas, creatina quinase-MB (CK-MB) e peptídeo natriurético tipo B (BNP) são incorporados à estratificação de risco, novos biomarcadores, incluindo troponina cardíaca de alta sensibilidade, pró-peptídeo natriurético tipo B N-terminal (NT-proBNP) e proteína C-reativa (PCR) estão atualmente sob exploração. A previsão de MACE permanece intrincada devido à natureza multifatorial do IAM e à variabilidade da resposta individual do paciente, necessitando de pesquisa contínua para modelos preditivos aprimorados.

O óxido nítrico (NO) e a endotelina-1 (ET-1) são os principais mediadores sintetizados pelas células endoteliais vasculares e desempenham papéis antagônicos.^11^ A ET-1 pode induzir constrição vascular, aumentar a atividade de adesão dos monócitos, levar à remodelação vascular e acelerar o desenvolvimento da aterosclerose.^12 , 13^ O NO tem a função de dilatar os vasos sanguíneos e inibir a agregação plaquetária, o que pode resistir à progressão da aterosclerose.^14^ Além disso, o NO pode inibir a síntese e a secreção de ET-1 pelas células endoteliais vasculares.^15^ A abertura da circulação colateral coronária pode aliviar significativamente os sintomas de isquemia miocárdica em pacientes com doença cardíaca coronária, o que está intimamente relacionado ao papel regulador do NO.

Aqui, objetivamos realizar um estudo retrospectivo para avaliar o valor preditivo de NO e ET-1 para estratificar riscos para MACE. Realizamos acompanhamentos de um ano de pacientes submetidos a PCI, que foram então categorizados em grupos daqueles que apresentaram MACE e aqueles que não apresentaram. As comparações são feitas entre os parâmetros basais (na admissão) de ambos os grupos. A análise é conduzida sobre a relação entre os níveis séricos de NO e ET-1 em pacientes com IAMCSST na admissão, a gravidade do IAMCSST e seu valor preditivo para a ocorrência de MACE dentro de um ano após PCI.

## Materiais e métodos

### Pacientes

O comitê de ética do Shengzhou People’s Hospital aprovou nosso estudo. Dados de pacientes admitidos durante 2018-2022 em nosso hospital foram usados com o critério de proteção de privacidade. Os seguintes critérios de inclusão e exclusão foram usados.

Critérios de inclusão

Idade ≥ 18 anos; início agudo do infarto do miocárdio até o tempo de admissão hospitalar <12 h; diagnóstico de IAMCSST de acordo com as “Diretrizes de Prática Clínica de Síndrome Coronariana Aguda de Emergência Chinesa de 2015”.^16^

### Critérios de exclusão

Foram excluídos pacientes com histórico de infarto do miocárdio antigo; pacientes com insuficiência cardíaca crônica; pacientes com hipertensão arterial pulmonar, doença cardíaca pulmonar ou insuficiência hepática e renal grave e doença craniana devido a várias causas; doença cardíaca congênita, doença cardíaca valvular, miocardite, histórico de pericardite; pacientes com tumor maligno; pacientes com histórico prévio de infarto do miocárdio ou insuficiência cardíaca; expectativa de vida estimada <1 ano.

O desfecho primário do MACE durante 1 ano de acompanhamento inclui principalmente readmissão devido a angina instável, novo início de insuficiência cardíaca aguda, IAM recorrente, choque cardiogênico, revascularização, arritmia grave (maligna) e morte. Angina instável é uma condição clínica entre angina estável e IAM, com subtipos incluindo angina de esforço de início recente, piora da angina de esforço, angina de repouso, angina pós-infarto e angina variante, cada uma caracterizada por sintomas e critérios diagnósticos específicos. O início recente de insuficiência cardíaca aguda é diagnosticado usando avaliações clínicas, de ECG, ecocardiográficas, laboratoriais e de biomarcadores, com uma classificação Killip de II-IV. IAM recorrente refere-se a um evento de IAM repetido. O choque cardiogênico é identificado por hipotensão persistente e sinais de perfusão inadequada de órgãos. A revascularização envolve passar por ICP ou CRM durante o acompanhamento de um ano após ICP. Arritmias graves que causam instabilidade hemodinâmica incluem fibrilação ventricular, taquicardia ventricular e outras, frequentemente levando à síncope ou morte súbita. A morte é definida como mortalidade durante o período de acompanhamento de um ano após ICP.

### Medição de NO sérico e ET-1

As concentrações séricas de NO e ET-1 foram quantificadas utilizando kits de ensaio imunoenzimático (ELISA) disponíveis comercialmente, de acordo com as instruções do fabricante (Thermo Fisher).

### Análise estatística

Variáveis contínuas com distribuição normal foram descritas usando média ± desvio padrão, e aquelas sem distribuição normal foram descritas usando mediana e intervalo interquartil. Variáveis categóricas foram expressas como frequências absolutas (n) e relativas (%). O teste-t de Student não pareado com correção de Welch ou o teste de Mann-Whitney foram usados para variáveis contínuas de acordo com a normalidade dos dados. A comparação entre variáveis categóricas foi feita usando o teste Qui-Quadrado ou Exato de Fisher. O teste de Kolmogorov-Smirnov foi usado para normalidade dos dados antes da análise. A análise de correlação de Pearson foi usada para analisar correlações entre dois parâmetros. A curva ROC ( *Receiver Operating Characteristic* ) foi usada para analisar os valores preditivos para MACE. A significância estatística foi determinada quando os valores de p foram menores que 0,05.

## Resultados

### Características de base

Um total de 269 pacientes foram incluídos no estudo ( Tabela 1 ). Após um ano de acompanhamento, 112 pacientes apresentaram eventos cardiovasculares adversos maiores (MACE) pós-procedimento, enquanto 157 pacientes não o apresentaram. Comparações das características basais dos pacientes na admissão entre esses dois grupos foram conduzidas, revelando diferenças significativas em idade, classificação de Killip, fração de ejeção do ventrículo esquerdo (FEVE), troponina cardíaca I (cTnI), creatina quinase-MB (CK-MB), bem como níveis séricos de NO e ET-1.

**Tabela 1:** – Características basais dos fatores clínicos para eventos cardiovasculares adversos maiores (MACE) pós-procedimento com início em um ano de acompanhamento entre pacientes com infarto agudo do miocárdio com elevação do segmento ST (IAMCSST)

Fatores	NMACE (n=157)	MACE (n=112)	Valor-p
**Idade (anos)**	61 (55,5, 68)	66 (58, 71,75)	0,005
**Índice de massa corporal (kg/m^2^)**	23 (21, 25,25)	24 (21, 25,5)	0.207
**Gênero (n, %)**			
Masculino	76 (48,4%)	61 (54,5%)	0,387
Feminino	81 (51,6%)	51 (45,5%)	
**Diabetes mellitus**			
Sim	22 (14%)	20 (17,9%)	0,399
Não	135 (86%)	92 (82,1%)	
**Hipertensão**			
Sim	34 (21,7%)	35 (31,3%)	0,089
Não	123 (78,3%)	77 (68,7%)	
**Hiperlipidemia**			
Sim	29 (18,5%)	28 (25%)	0,227
Não	128 (81,5%)	84 (75%)	
**Tabagismo**			
Sim	58 (36,9%)	49 (43,8%)	0,312
Não	99 (63,1%)	63 (56,2%)	
**Classe Killip na admissão (n, %)**			
1	71 (45,2%)	27 (24,1%)	0,001
2	36 (22,9%)	25 (22,3%)	
3	29 (18,5%)	36 (32,2%)	
4	21 (13,4%)	24 (21,4%)	
**Frequência cardíaca (bpm)**	78 (72,5, 86)	81 (75, 86)	0,117
**PAS (mmHg)**	127,63 ± 19,15	130,73 ± 20,71	0,174
**PAD (mmHg)**	85,34 ± 11,27	88,66 ± 10,92	0,218
**FEVE (%)**	51,92 (46,78, 56,27)	46,42 (42,21, 50,45)	< 0,001
**cTnI sérico (ng/mL)**	0,87 ± 0,21	1,36 ± 0,45	< 0,001
**CK-MB sérico (ng/mL)**	28,54 ± 8,11	40,65 ± 12,19	< 0,001
**NO sérico (nmol/mL)**	35,45 ± 9,51	28,77 ± 9,15	< 0,001
**ET-1 séric (pg/mL)**	64,56 ± 15,57	80,95 ± 18,85	< 0,001

As variáveis contínuas foram apresentadas como média ± DP quando atendiam à distribuição normal ou como mediana (intervalo interquartil) quando não atendiam à distribuição normal. As variáveis categóricas foram apresentadas como n (porcentagem). NMACE: nenhum evento cardiovascular adverso maior; PAS: pressão arterial sistólica; PAD: pressão arterial diastólica; FEVE: fração de ejeção do ventrículo esquerdo; cTnI: troponina I cardíaca; CK-MB: creatina quinase-músculo/cérebro.

### Comparação dos níveis séricos de NO e ET-1 entre grupos de pacientes

**Figura 1:**
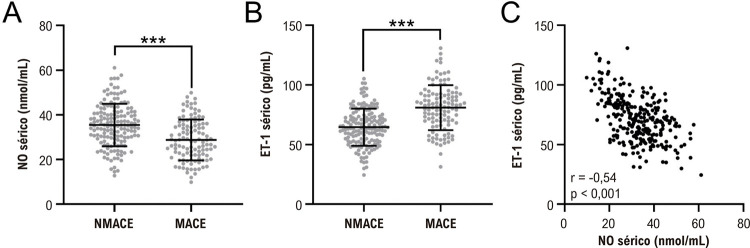
– Comparações de NO sérico (A) e ET-1 (B) na admissão entre pacientes com (n=112) e sem (n=156) eventos cardiovasculares adversos maiores (MACE) pós-procedimento em um ano após infarto agudo do miocárdio com elevação do segmento ST (IAMCSST). ***p < 0,001. Teste t não pareado com correção de Welch. C, Análise de correlação de Pearson de NO sérico com ET-1 na admissão em pacientes com infarto agudo do miocárdio com elevação do segmento ST (IAMCSST, n = 269).

A Figura 1 compara os níveis séricos de NO e ET-1 na admissão entre os 112 pacientes que apresentaram MACE e os 157 pacientes que não apresentaram. Os resultados mostram que os pacientes que apresentaram MACE apresentaram níveis séricos mais baixos de NO ( Figura 1A ) e mais altos de ET-1 ( Figura 1B ) na admissão em comparação com aqueles que não apresentaram (p<0,001). Além disso, uma correlação negativa significativa foi observada entre os níveis séricos de NO e ET-1 entre todos os pacientes com IAMCSST ( Figura 1C ).

### Relação entre os níveis séricos de NO e ET-1 e a gravidade do IAMCSST

**Figura 2:**
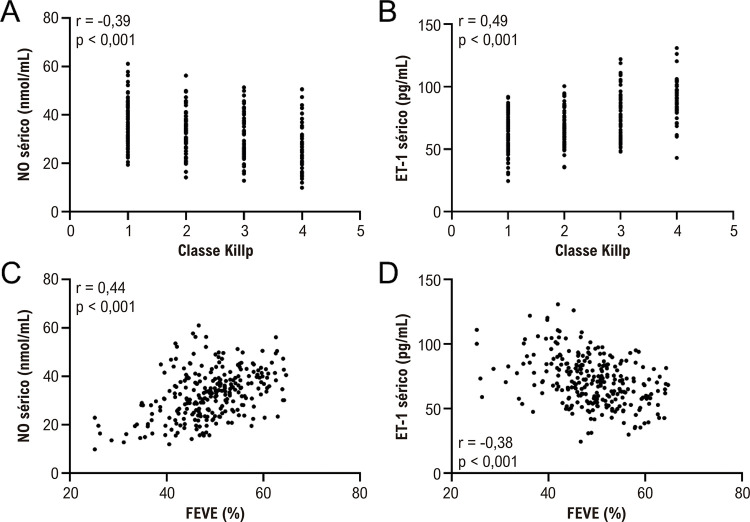
– Análise de correlação de Spearman da classe Killp com NO sérico (A) e ET-1 (B) na admissão em pacientes com infarto agudo do miocárdio com supradesnivelamento do segmento ST (IAMCSST, n = 269). Análise de correlação de Spearman da FEVE com NO sérico (C) e ET-1 (D) na admissão em pacientes com infarto agudo do miocárdio com supradesnivelamento do segmento ST (IAMCSST, n = 269).

Em seguida, analisamos a relação entre os níveis séricos de NO e ET-1 na admissão e a gravidade do IAMCSST em todos os pacientes ( Figura 2 ). A classe Killip e a FEVE podem refletir a gravidade do infarto do miocárdio em pacientes com IAMCSST. Pode-se observar que quanto mais grave o infarto do miocárdio em pacientes com IAMCSST, menor o NO sérico ( Figura 1C e 2C ) e maior os níveis de ET-1 ( Figura 2B e 2D ) na admissão, com correlações positivas e negativas marcantes, respectivamente.

### Valor preditivo dos níveis séricos de NO e ET-1 para MACE

**Figura 3:**
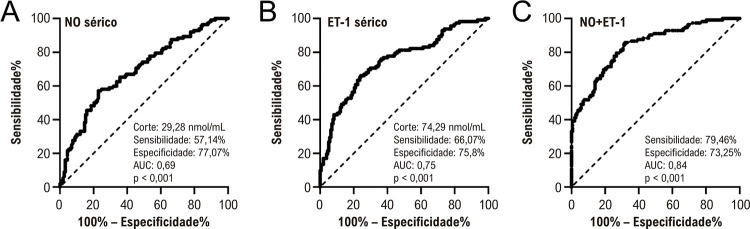
– Análise ROC de NO sérico (A), ET-1 (B) na admissão e seu teste combinado (C) para predição de eventos cardiovasculares adversos maiores (MACE) com início em um ano de acompanhamento entre pacientes com infarto agudo do miocárdio com elevação do segmento ST (IAMCSST).

Para demonstrar o valor preditivo do NO sérico e ET-1, a análise ROC foi realizada para usar NO único ( Figura 3A ) ou ET-1 ( Figura 3B ) ou seu valor combinado ( Figura 3C ) para prever a ocorrência de MACE dentro de um ano após ICP em pacientes com IAMCSST. Os valores de corte, sensibilidade e especificidade da análise ROC foram determinados com base no valor máximo do índice de Youden, conforme visto nos dados da figura e na área sob a curva. O modelo de detecção combinado foi representado pela fórmula: -0,082 * NO + 0,059 * ET-1

### Classificação de pacientes com IAMCSST com base no modelo de detecção combinado

Em seguida, dividimos todos os pacientes com IAMCSST em grupos alto (> 1,445, n=131) e baixo (< 1,445, n=138) com base nos valores de corte na análise ROC do modelo de detecção combinado dos níveis séricos de NO e ET-1 na admissão ( Tabela 2 ). Nenhum dos pacientes apresentou valor exatamente neste valor de corte de 1,445. Durante um acompanhamento de um ano após a ICP, houve 1 caso (grupo < 1,445) e 3 casos (grupo > 1,445) de choque cardiogênico ( Tabela 2 ), todos fatais. Houve 6 casos e 16 casos com arritmia maligna no grupo < 1,445 e no grupo > 1,455, respectivamente. Uma diferença significativa foi observada no caso de insuficiência cardíaca (grupo < 1,455: 11 casos; grupo > 1,455: 27 casos). Não houve diferença significativa nos casos de infarto do miocárdio recorrente ou angina recorrente que necessitou de revascularização. Além disso, a mortalidade de acompanhamento de um ano foi de 2 casos (grupo < 1,445) e 8 casos (grupo > 1,445), todos os quais foram classificados como mortalidade cardíaca, sem outras causas de morte registradas. A comparação da ocorrência de MACE pós-procedimento entre os dois grupos revelou diferenças significativas em todas as comparações, ilustrando ainda mais o valor preditivo promissor do modelo de detecção combinado.

**Tabela 2:** – Comparações de eventos cardiovasculares adversos maiores (MACE) pós-procedimento com início em um ano de acompanhamento entre pacientes com infarto agudo do miocárdio com supradesnivelamento do segmento ST (IAMCSST) de acordo com o ponto de corte do teste de combinação de NO sérico e ET-1 (-0,082 * NO + 0,059 * ET-1)

	< 1,445 (n=138)	> 1,445 (n=131)	Valor-p
Arritmia maligna	6 (4,3%)	16 (12,2%)	0,025
Choque cardiogênico	1 (0,7%)	3 (2,3%)	0,359
Insuficiência cardíaca	11 (8,0%)	27 (20,6%)	0,005
Infarto do miocárdio recorrente	7 (5,1%)	12 (9,2%)	0,237
Angina recorrente que requer revascularização	8 (5,8%)	15 (11,4%)	0,129
Óbito	2 (1,4%)	8 (6,1%)	0,055
MACE Total	34 (24,6%)	78 (59,5%)	< 0,001

MACE: eventos cardiovasculares adversos maiores.

## Discussão

O estudo atual examina as implicações prognósticas dos níveis séricos de NO e ET-1 na predição de MACE em pacientes com IAMCSST que foram submetidos a ICP. Consistente com as alterações cardiológicas causadas por MACE, disparidades significativas foram identificadas em parâmetros-chave como classificação de Killip, FEVE, cTnI, CK-MB e, particularmente, os níveis séricos de NO e ET-1 entre pacientes que apresentaram MACE e aqueles que não apresentaram, após ICP.^17^ A maior média de idade do grupo MACE sugeriu que a idade é um fator importante associado ao alto risco de MACE.^18^ Em consonância com pesquisas anteriores, nosso estudo revelou uma correlação inversa entre os níveis séricos de NO e ET-1,^19^ ou seja, pacientes que apresentaram MACE apresentaram níveis significativamente mais baixos de NO e níveis mais altos de ET-1 na admissão em comparação com aqueles sem MACE, enfatizando seus papéis antagônicos. Essa descoberta ressalta a relação fisiopatológica entre a função endotelial prejudicada, manifestada por níveis mais baixos de NO e mais altos de ET-1, e piores resultados cardiovasculares em pacientes com IAMCSST. O NO, um vasodilatador, inibe a agregação plaquetária e resiste à progressão aterosclerótica, enquanto a ET-1 induz vasoconstrição e remodelação vascular, acelerando o desenvolvimento da aterosclerose.

Nossos achados também indicam uma associação distinta entre a gravidade do IAMCSST, representada pela classe Killip e FEVE, e os níveis séricos de NO e ET-1. Quanto mais grave o infarto do miocárdio, menor o NO sérico e maior os níveis de ET-1 na admissão, o que se alinha com nossa compreensão dos processos fisiopatológicos do infarto do miocárdio. Além disso, nossa análise ROC demonstra um valor preditivo convincente dos níveis séricos de NO e ET-1 na admissão para a ocorrência de MACE dentro de um ano após a ICP. Notavelmente, o modelo de detecção combinado dos níveis de NO e ET-1 parece aumentar esse poder preditivo, conforme representado por uma diferença significativa na incidência de MACE pós-procedimento entre os grupos alto e baixo com base no valor de corte do modelo de detecção combinado. Esses dados apoiam o uso dos níveis séricos de NO e ET-1 como uma ferramenta de diagnóstico para o tratamento de pacientes com IAMCSST, estratificando seu risco para MACE. Essa abordagem foi testada anteriormente para outras doenças miocárdicas.^20 , 21^ O uso de biomarcadores séricos é mais acessível e econômico do que os exames de imagem e mais objetivo do que os sistemas de pontuação clínica.

Embora nosso estudo comprove o valor clínico dos níveis séricos de NO e ET-1 na previsão de resultados pós-ICP, ele também acentua a importância da avaliação rápida e abrangente desses biomarcadores em pacientes com IAMCSST. Essas descobertas estabelecem uma base para potenciais estratégias terapêuticas visando a modulação das vias de NO e ET-1.

O estudo atual tem várias limitações que devem ser reconhecidas. Primeiro, a população do estudo é relativamente pequena e confinada a um único centro, o que pode limitar a generalização dos achados para populações e cenários mais amplos. A natureza retrospectiva da análise também pode introduzir viés de seleção, pois a inclusão de pacientes que já foram submetidos a ICP pode distorcer os resultados para aqueles com apresentações mais graves de IAMCSST. Além disso, o estudo incluiu apenas pacientes que sobreviveram para serem internados no hospital, potencialmente excluindo aqueles com as manifestações mais graves de IAMCSST. O período de acompanhamento de um ano, embora significativo, pode não capturar complicações pós-procedimento de início tardio, e o impacto da sobrevivência a longo prazo e da qualidade de vida permanece inexplorado. Além disso, o estudo não considera variações no tratamento médico pós-ICP, o que pode influenciar os resultados e a ocorrência de MACE. Finalmente, embora tenham sido observadas correlações significativas entre os níveis séricos de NO e ET-1 com a gravidade do IAMCSST e MACE, a causalidade não pode ser estabelecida, e outros fatores de confusão não medidos podem influenciar esses biomarcadores e resultados clínicos. Mais estudos são necessários com um desenho multicêntrico, tamanhos de amostra maiores e um acompanhamento mais longo para entender completamente o valor preditivo desses biomarcadores. Mais pesquisas sobre os mecanismos fisiopatológicos de NO e ET-1 em IAMCSST e estudos longitudinais mais extensos sobre as implicações clínicas desses marcadores são garantidos.

## Conclusões

Em conclusão, este estudo ressalta o papel fundamental dos níveis séricos de NO e ET-1 na predição de MACE em pacientes com IAMCSST submetidos a ICP ( Figura Central ). Notavelmente, nossas descobertas elucidam uma clara correlação inversa entre os níveis séricos de NO e ET-1 e a gravidade do infarto do miocárdio. Mais importante, o modelo de detecção combinado de NO e ET-1 aumenta significativamente o poder prognóstico para a ocorrência de MACE dentro de um ano após a ICP. Essas descobertas sugerem que os níveis séricos de NO e ET-1 podem servir como biomarcadores valiosos para estratificação de risco e tomada de decisão terapêutica em pacientes com IAMCSST submetidos a ICP. No entanto, a aplicação clínica dessas descobertas requer validação por meio de mais estudos prospectivos e multicêntricos. Os mecanismos fisiopatológicos subjacentes de NO e ET-1 no contexto do IAMCSST também justificam uma investigação mais aprofundada.
