# Supplementary material for: Óxido Nítrico Sérico, Endotelina-1 Correlaciona Eventos Cardiovasculares Adversos Importantes Pós-Procedimento entre Pacientes com IAMCSST Agudo
Source: Arq Bras Cardiol. 2024 Dec 16;122(1):e20240248. [Article in Portuguese] doi: 10.36660/abc.20240248 (PMC11834733; doi:10.36660/abc.20240248)
Supplement: Translation

# Translation: Serum Nitric Oxide, Endothelin-1 Correlates Post-Procedural Major Adverse Cardiovascular Events among Patients with Acute STEMI

**DOI:** 10.36660/abc.20240248i

**Authors:** Huihui Guo, Qi Qu, Jiechao Lv

**Affiliations:** 1 China Department of Cardiovascular Medicine - Shengzhou People’s Hospital (Shengzhou Branch of the First Affiliated Hospital of Zhejiang University School of Medicine, the Shengzhou Hospital of Shaoxing University), Zhejiang – China

**Keywords:** ST Elevation Myocardial Infarction, Myocardial Infarction, Prognosis

## Abstract

**Background:**

ST-segment elevation myocardial infarction (STEMI) is a common and severe form of acute myocardial infarction (AMI).

**Objectives:**

The study aimed to investigate the relationship between serum nitric oxide (NO) and endothelin-1 (ET-1) levels with the severity of STEMI and their predictive value for major adverse cardiovascular events (MACE) within one year after percutaneous coronary intervention (PCI) in STEMI patients.

**Methods:**

The retrospective study was conducted on 269 STEMI patients who underwent PCI. The patients were categorized into two groups: those who developed MACE (112 cases) and those who did not (157 cases) within one year. NO and ET1 levels were measured in collected serum using enzyme-linked immunosorbent assay. Receive-operating characteristics (ROC) curve was used to analyze the prognostic potential of NO and ET1 individually and in combination, p<0.05 was considered statistically significant.

**Results:**

Significant differences were noted between the two groups concerning age, Killip classification, left ventricular ejection fraction, cardiac troponin I (cTnI), creatine kinase-MB (CK-MB), as well as serum NO and ET-1 levels. The study observed that patients who developed MACE had lower serum NO and higher ET-1 levels upon admission. Further analysis revealed a significant inverse relationship between serum NO and ET-1 levels and the severity of myocardial infarction. A combined detection model, -0.082 * NO + 0.059 * ET-1, demonstrated promising prognostic value for the occurrence of MACE within one year post-PCI.

**Conclusions:**

Serum NO and ET-1 levels serve as valuable prognostic markers for MACE in STEMI patients undergoing PCI, exhibiting a strong correlation with AMI severity.

## Introduction

Acute myocardial infarction (AMI) is a common cardiac disease in cardiology, characterized by the sudden reduction or interruption of blood flow due to coronary artery obstruction,^1^leading to myocardial hypoxia, ischemia, necrosis, and subsequent complications such as chest pain and arrhythmias.^2 , 3^The disease onset is acute, with rapid progression, and can pose a severe risk to the patient’s life if not promptly intervened. AMI is categorized into types such as ST-segment elevation myocardial infarction (STEMI), Q-wave, and dynamic ST-T changes, with STEMI being the most prevalent.^4^The wide application of emergency percutaneous coronary intervention (PCI) has significantly reduced the mortality rate of STEMI patients.^5 , 6^However, prolonged ischemia and ischemia-reperfusion injury can cause myocardial cell death, leading to major cardiovascular adverse events (MACE) in the short term after myocardial infarction, severely affecting patient prognosis. Therefore, markers of STEMI prognosis are critical in reducing the area of myocardial infarction, preventing left ventricular remodeling, reducing the occurrence of MACE, and ultimately improving the prognosis of STEMI patients.

Current risk stratification strategies for MACE encompass clinical scoring systems (e.g., GRACE, TIMI, PURSUIT scores),^7^imaging modalities (echocardiography, coronary angiography, cardiac magnetic resonance imaging (MRI), computed tomography (CT) angiography),^8 , 9^and biomarker-based approaches.^10^While traditional cardiac biomarkers such as cardiac troponins, creatine kinase-MB (CK-MB), and B-type natriuretic peptide (BNP) are incorporated into risk stratification, novel biomarkers including high-sensitivity cardiac troponin, N-terminal pro-b-type natriuretic peptide (NT-proBNP), and C-reactive protein (CRP) are currently under exploration. Predicting MACE remains intricate due to the multifactorial nature of AMI and individual patient response variability, necessitating ongoing research for improved predictive models.

Nitric oxide (NO) and Endothelin-1 (ET-1) are the main mediators synthesized by vascular endothelial cells and play antagonistic roles.^11^ET-1 can induce vascular constriction, enhance the adhesion activity of monocytes, lead to vascular remodeling, and accelerate the development of atherosclerosis.^12 , 13^NO has the function of dilating blood vessels and inhibiting platelet aggregation, which can resist the progression of atherosclerosis.^14^Furthermore, NO can inhibit the synthesis and secretion of ET-1 by vascular endothelial cells.^15^The opening of the coronary collateral circulation can significantly alleviate the symptoms of myocardial ischemia in patients with coronary heart disease, which is closely related to the regulatory role of NO.

Herein, we aimed to perform a retrospective study to evaluate the predictive value of NO and ET-1 for stratifying risks for MACE. We conducted one-year follow-ups of patients who underwent PCI, who were then categorized into groups of those who experienced MACE and those who did not. Comparisons are made between the baseline parameters (at admission) of both groups. The analysis is conducted on the relationship between the serum levels of NO and ET-1 in STEMI patients at admission, the severity of STEMI, and their predictive value for the occurrence of MACE within one year after PCI.

## Materials and Methods

### Patients

The ethical committee of Shengzhou People’s Hospital approved our study. Data of patients admitted during 2018-2022 to our hospital were used with the discretion of privacy protection. The following inclusion and exclusion criteria were used.

### Inclusion criteria

Age ≥ 18 years; acute onset of myocardial infarction to hospital admission time <12 h; diagnosis of STEMI according to the “2015 Chinese Emergency Acute Coronary Syndrome Clinical Practice Guidelines Diagnosis Standards”.^16^

### Exclusion criteria

Patients with a history of old myocardial infarction; patients with chronic heart failure; patients with pulmonary artery hypertension, pulmonic heart disease or severe liver and kidney function insufficiency and cranial disease due to various causes; congenital heart disease, valvular heart disease, myocarditis, pericarditis history; malignant tumor patients; patients with a previous history of myocardial infarction or heart failure; estimated life expectancy <1 year were excluded.

The primary endpoint of MACE during 1 year of follow-up mainly includes readmission due to unstable angina, new onset of acute heart failure, recurrent AMI, cardiogenic shock, revascularization, severe (malignant) arrhythmia, and death. Unstable angina is a clinical condition between stable angina and acute myocardial infarction (AMI), with subtypes including new-onset exertional angina, worsening exertional angina, rest angina, post-infarction angina, and variant angina, each characterized by specific symptoms and diagnostic criteria. New onset of acute heart failure is diagnosed using clinical, ECG, echocardiography, laboratory, and biomarker evaluations, with a Killip classification of II-IV. Recurrent AMI refers to a repeat AMI event. Cardiogenic shock is identified by persistent hypotension and inadequate organ perfusion signs. Revascularization involves undergoing PCI or CABG during the one-year follow-up after PCI. Severe arrhythmias causing hemodynamic instability include ventricular fibrillation, ventricular tachycardia, and others, often leading to syncope or sudden death. Death is defined as mortality during the one-year follow-up period post-PCI.

### Measurement of serum NO and ET-1

Serum NO and ET-1 concentrations were quantified utilizing commercially available enzyme-linked immunosorbent assay (ELISA) kits in accordance with the manufacturer’s instructions (Thermo Fisher).

### Statistical analysis

Continuous variables with normal distribution were described using mean ± standard deviation, and those without normal distribution were described using median and interquartile range. Categorical variables were expressed as absolute (n) and relative (%) frequencies. The unpaired Student’s t-test with Welch’s correction or the Mann-Whitney test were used for continuous variables according to data normality. The comparison between categorical variables was made using the Chi-Square or Fisher’s Exact test. The Kolmogorov-Smirnov test was used normality of the data before analysis. Pearson correlation analysis was used to analyze correlations between two parameters. The receiver operating characteristic (ROC) curve was used to analyze the predictive values for MACE. Statistical significance was determined when p-values were less than 0.05.

## Results

### Baseline characteristics

A total of 269 patients were included in the study ( Table 1 ). After one year of follow-up, 112 patients experienced major adverse cardiovascular events (MACE) post-procedurally, while 157 patients did not. Comparisons of the baseline characteristics of patients upon admission between these two groups were conducted, revealing significant differences in age, Killip classification, left ventricular ejection fraction (LVEF), cardiac troponin I (cTnI), creatine kinase-MB (CK-MB), as well as serum levels of NO and ET-1.

**Table 1:** – Baseline characteristics of clinical factors for post-procedural major adverse cardiovascular events (MACE) onset at one-year following-up among patients with acute ST-segment elevation myocardial infarction (STEMI)

Factors	NMACE (n=157)	MACE (n=112)	p-value
**Age (years)**	61 (55.5, 68)	66 (58, 71.75)	0.005
**Body mass index (kg/m^2^)**	23 (21, 25.25)	24 (21, 25.5)	0.207
**Gender (n, %)**			
Male	76 (48.4%)	61 (54.5%)	0.387
Female	81 (51.6%)	51 (45.5%)	
**Diabetes mellitus**			
Yes	22 (14%)	20 (17.9%)	0.399
No	135 (86%)	92 (82.1%)	
**Hypertension**			
Yes	34 (21.7%)	35 (31.3%)	0.089
No	123 (78.3%)	77 (68.7%)	
**Hyperlipidemia**			
Yes	29 (18.5%)	28 (25%)	0.227
No	128 (81.5%)	84 (75%)	
**Smoking**			
Yes	58 (36.9%)	49 (43.8%)	0.312
No	99 (63.1%)	63 (56.2%)	
**Killip class on admission (n, %)**			
1	71 (45.2%)	27 (24.1%)	0.001
2	36 (22.9%)	25 (22.3%)	
3	29 (18.5%)	36 (32.2%)	
4	21 (13.4%)	24 (21.4%)	
**Heart rate (b.p.m.)**	78 (72.5. 86)	81 (75. 86)	0.117
**SBP (mmHg)**	127.63 ± 19.15	130.73 ± 20.71	0.174
**DBP (mmHg)**	85.34 ± 11.27	88.66 ± 10.92	0.218
**LVEF (%)**	51.92 (46.78, 56.27)	46.42 (42.21, 50.45)	< 0.001
**Serum cTnI (ng/mL)**	0.87 ± 0.21	1.36 ± 0.45	< 0.001
**Serum CK-MB (ng/mL)**	28.54 ± 8.11	40.65 ± 12.19	< 0.001
**Serum NO (nmol/mL)**	35.45 ± 9.51	28.77 ± 9.15	< 0.001
**Serum ET-1 (pg/mL)**	64.56 ± 15.57	80.95 ± 18.85	< 0.001

The continuous variables were presented as mean ± SD when they met normal distribution or as median (interquartile range) when they did not meet normal distribution. The categorical variables were presented as n (percentage). NMACE: no major adverse cardiovascular events; SBP: systolic blood pressure; DBP: diastolic blood pressure; LVEF: left ventricular ejection fraction; cTnI: cardiac troponin I; CK-MB: creatine kinase-muscle/brain.

### Comparison of Serum NO and ET-1 levels between patient groups

Figure 1 compares the serum levels of NO and ET-1 upon admission between the 112 patients who experienced MACE and the 157 patients who did not. The results show that patients who experienced MACE had lower serum NO ( Figure 1A ) and higher ET-1 ( Figure 1B ) levels upon admission compared to those who did not (p<0.001). Furthermore, a significant negative correlation was observed between serum NO and ET-1 levels among all STEMI patients ( Figure 1C ).

**Figure 1:** – Comparisons of serum NO (A) and ET-1 (B) at admission between patients with (n=112) and without (n=156) post-procedural major adverse cardiovascular events (MACE) onset at one-year following up acute ST-segment elevation myocardial infarction (STEMI). ***p < 0.001. Unpaired t-test with Welch’s correction. (C) Pearson correlation analysis of serum NO with ET-1 at admission in patients with acute ST-segment elevation myocardial infarction (STEMI, n = 269).

### Relationship between Serum NO and ET-1 levels and stemi severity

We next analyzed the relationship between serum levels of NO and ET-1 upon admission and the severity of STEMI in all patients ( Figure 2 ). The Killip class and LVEF can reflect the severity of myocardial infarction in STEMI patients. It can be seen that the more severe the myocardial infarction in STEMI patients, the lower the serum NO ( Figure 2A and 2C ) and the higher the ET-1 levels ( Figure 2B and 2D ) upon admission, with marked positive and negative correlations, respectively.

**Figure 2:** – Spearman correlation analysis of Killp class with serum NO (A) and ET-1 (B) at admission in patients with acute ST-segment elevation myocardial infarction (STEMI, n = 269). Spearman correlation analysis of LVEF with serum NO (C) and ET-1 (D) at admission in patients with acute ST-segment elevation myocardial infarction (STEMI, n = 269).

### Predictive value of Serum NO and ET-1 levels for MACE

To demonstrate the predictive value of serum NO and ET-1, ROC analysis was performed to use single NO ( Figure 3A ) or ET-1 ( Figure 3B ) or their combined value ( Figure 3C ) to predict the occurrence of MACE within one year after PCI in STEMI patients. The cut-off values, sensitivity, and specificity of the ROC analysis were determined based on the maximum value of the Youden index, as seen in the figure data and area under the curve. The combined detection model was represented by the formula: -0.082 * NO + 0.059 * ET-1.

**Figure 3:** – ROC analysis of serum NO (A), ET-1 (B) at admission and their combined test (C) for prediction of major adverse cardiovascular events (MACE) onset at one-year following up among patients with acute ST-segment elevation myocardial infarction (STEMI).

### Classification of STEMI patients based on the combined detection model

We next divided all STEMI patients into high (> 1.445, n=131) and low (< 1.445, n=138) groups based on the cut-off values in the ROC analysis of the combined detection model of serum NO and ET-1 levels upon admission ( Table 2 ). None of the patients had value exactly at this cut-off value of 1.445. During a one-year follow-up after PCI, there were 1 case (< 1.445 group) and 3 cases (> 1.445 group) of cardiogenic shock ( Table 2 ), all of which were fatal. There were 6 cases and 16 cases with malignant arrhythmia in the < 1.445 group and > 1.455 group, respectively. A significant difference was observed in the case of heart failure (< 1.455 group: 11 cases; > 1.455 group: 27 cases). There was no significant difference in the cases of recurrent myocardial infarction or recurrent angina requiring revascularization. Additionally, the one-year follow-up mortality was 2 cases (< 1.445 group) and 8 cases (> 1.445 group), all of which were classified as cardiac mortality, with no other causes of death recorded. The comparison of the occurrence of MACE post-procedurally between the two groups revealed significant differences in all comparisons, further illustrating the promising predictive value of the combined detection model.

**Table 2:** –Comparisons of post-procedural major adverse cardiovascular events (MACE) onset at one-year following up among patients with acute ST-segment elevation myocardial infarction (STEMI) according to the cut-off of serum NO and ET-1 combination test (-0.082 * NO + 0.059 * ET-1)

	< 1,445 (n=138)	> 1,445 (n=131)	p-value
Malignant arrhythmia	6 (4.3%)	16 (12.2%)	0.025
Cardiogenic shock	1 (0.7%)	3 (2.3%)	0.359
Heart failure	11 (8.0%)	27 (20.6%)	0.005
Recurrent myocardial infarction	7 (5.1%)	12 (9.2%)	0.237
Recurrent angina requiring revascularization	8 (5.8%)	15 (11.4%)	0.129
Death	2 (1.4%)	8 (6.1%)	0.055
Total MACE	34 (24.6%)	78 (59.5%)	< 0.001

## Discussion

The current study scrutinizes the prognostic implications of serum NO and ET-1 levels in predicting MACE in patients with STEMI who have undergone PCI. Consistent with cardiological changes caused by MACE, significant disparities were identified in key parameters such as Killip classification, LVEF, cTnI, CK-MB, and particularly the serum levels of NO and ET-1 between patients who experienced MACE and those who did not, post-PCI.^17^The greater mean age of the MACE group suggested that age is an important factor associated with high MACE risk.^18^In line with previous research, our study revealed an inverse correlation between serum NO and ET-1 levels,^19^i.e., patients who experienced MACE had significantly lower NO levels and higher ET-1 levels upon admission compared to those without MACE, emphasizing their antagonistic roles. This finding underscores the pathophysiological relationship between impaired endothelial function, manifested by lower NO and higher ET-1 levels, and worse cardiovascular outcomes in STEMI patients. NO, a vasodilator, inhibits platelet aggregation and resists atherosclerotic progression, while ET-1 induces vasoconstriction and vascular remodeling, accelerating atherosclerosis development.

Our findings also indicate a distinct association between the severity of STEMI, represented by Killip class and LVEF, and the serum NO and ET-1 levels. The more severe the myocardial infarction, the lower the serum NO and the higher the ET-1 levels upon admission, which aligns with our understanding of the pathophysiological processes of myocardial infarction. Furthermore, our ROC analysis demonstrates a compelling predictive value of serum NO and ET-1 levels upon admission for the occurrence of MACE within one year post-PCI. Remarkably, the combined detection model of NO and ET-1 levels appears to enhance this predictive power, as depicted by a significant difference in the incidence of post-procedural MACE between the high and low groups based on the cut-off value of the combined detection model. These data support the use of serum NO and ET-1 levels as a diagnostic tool for managing patients with STEMI by stratifying their risk for MACE. This approach has been previously tested for other myocardial diseases.^20 , 21^The use of the serum biomarkers is more accessible and cost-effective than imaging and more objective than clinical scoring systems.

While our study substantiates the clinical value of serum NO and ET-1 levels in prognosticating post-PCI outcomes, it also accentuates the importance of prompt and comprehensive assessment of these biomarkers in STEMI patients. These findings lay a foundation for potential therapeutic strategies targeting the modulation of NO and ET-1 pathways.

The current study has several limitations that should be acknowledged. Firstly, the study population is relatively small and confined to a single center, which may limit the generalizability of the findings to broader populations and settings. The retrospective nature of the analysis might also introduce selection bias, as the inclusion of patients who have already undergone PCI could skew the results towards those with more severe STEMI presentations. Furthermore, the study only included patients who survived to be admitted to the hospital, potentially excluding those with the most severe manifestations of STEMI. The follow-up period of one year, while significant, may not capture late-onset post-procedural complications, and the impact of long-term survival and quality of life remains unexplored. Additionally, the study does not account for variations in medical treatment post-PCI, which could influence outcomes and the occurrence of MACE. Finally, while significant correlations between serum NO and ET-1 levels with STEMI severity and MACE were observed, causation cannot be established, and other unmeasured confounding factors could influence these biomarkers and clinical outcomes. Further studies are needed with a multicenter design, larger sample sizes, and a longer follow-up to understand the predictive value of these biomarkers fully. Further research into the pathophysiological mechanisms of NO and ET-1 in STEMI, and more extensive longitudinal studies on the clinical implications of these markers are warranted.

## Conclusions

In conclusion, this study underscores the pivotal role of serum NO and ET-1 levels in predicting MACE in patients with STEMI who undergo PCI ( Central Figure ). Notably, our findings elucidate a clear inverse correlation between serum NO and ET-1 levels and the severity of myocardial infarction. More importantly, the combined detection model of NO and ET-1 significantly enhances the prognostic power for the occurrence of MACE within one year post-PCI. These findings suggest that serum NO and ET-1 levels could serve as valuable biomarkers for risk stratification and therapeutic decision-making in STEMI patients undergoing PCI. However, the clinical application of these findings requires validation through further prospective, multicenter trials. The underlying pathophysiological mechanisms of NO and ET-1 in the context of STEMI also warrant further investigation.
